# Rat model of metastatic breast cancer monitored by MRI at 3 tesla and bioluminescence imaging with histological correlation

**DOI:** 10.1186/1479-5876-7-88

**Published:** 2009-10-20

**Authors:** Ho-Taek Song, Elaine K Jordan, Bobbi K Lewis, Wei Liu, Justin Ganjei, Brenda Klaunberg, Daryl Despres, Diane Palmieri, Joseph A Frank

**Affiliations:** 1 Frank Laboratory, Radiology and Imaging Sciences Clinical Center, National Institute of Health, Bethesda, MD, USA; 2 Department of Radiology, College of Medicine, Yonsei University, Seoul, Korea; 3 Philips Research North America, Briarcliff Manor, NY, USA; 4 Mouse Imaging Facility, National Institute of Neurological Disorder and Stroke, National Institute of Health, Bethesda, MD, USA; 5 Women's Cancers Section, Laboratory of Molecular Pharmacology, National Cancer Institute, National Institute of Health, Bethesda, MD, USA; 6 Intramural Research Program, National Institute of Biomedical Imaging and Bioengineering, 6120 Executive Blvd Bethesda, MD 20892, USA

## Abstract

**Background:**

Establishing a large rodent model of brain metastasis that can be monitored using clinically relevant magnetic resonance imaging (MRI) techniques is challenging. Non-invasive imaging of brain metastasis in mice usually requires high field strength MR units and long imaging acquisition times. Using the brain seeking MDA-MB-231BR transfected with luciferase gene, a metastatic breast cancer brain tumor model was investigated in the nude rat. Serial MRI and bioluminescence imaging (BLI) was performed and findings were correlated with histology. Results demonstrated the utility of multimodality imaging in identifying unexpected sights of metastasis and monitoring the progression of disease in the nude rat.

**Methods:**

Brain seeking breast cancer cells MDA-MB-231BR transfected with firefly luciferase (231BRL) were labeled with ferumoxides-protamine sulfate (FEPro) and 1-3 × 10^6 ^cells were intracardiac (IC) injected. MRI and BLI were performed up to 4 weeks to monitor the early breast cancer cell infiltration into the brain and formation of metastases. Rats were euthanized at different time points and the imaging findings were correlated with histological analysis to validate the presence of metastases in tissues.

**Results:**

Early metastasis of the FEPro labeled 231BRL were demonstrated onT2*-weighted MRI and BLI within 1 week post IC injection of cells. Micro-metastatic tumors were detected in the brain on T2-weighted MRI as early as 2 weeks post-injection in greater than 85% of rats. Unexpected skeletal metastases from the 231BRL cells were demonstrated and validated by multimodal imaging. Brain metastases were clearly visible on T2 weighted MRI by 3-4 weeks post infusion of 231BRL cells, however BLI did not demonstrate photon flux activity originating from the brain in all animals due to scattering of the photons from tumors.

**Conclusion:**

A model of metastatic breast cancer in the nude rat was successfully developed and evaluated using multimodal imaging including MRI and BLI providing the ability to study the temporal and spatial distribution of metastases in the brain and skeleton.

## Background

The most common tumors in the central nervous system are metastasis originating from lung and breast cancer [1,2]. Brain metastasis occurs in 51% of breast cancer patients, with a median survival of 13 months despite the institution of early treatment [1,3,4]. Magnetic resonance imaging (MRI) is a sensitive diagnostic tool with high spatial resolution and excellent tissue contrast used to detect brain metastases in patients with breast cancer [5,6] however, MRI alone cannot identify micro-metastases or track dormant malignant cells in the brain [4,7,8]. Yoneda et al derived the MDA-MB-231BR cell line (231BR) that is estrogen independent from metastatic ductal carcinoma [9,10] and specifically homes to brain [11]. Intracardiac (IC) injection of the 231BR cells in nude mice was reported to form metastases in the brain and therefore animal models based on this cell line have been used for this purpose [8,12,13]. Recently, the 231BR was transfected with enhanced green fluorescent protein (EGFP) and following IC injection in mice and metastases were detected brains by optical imaging and fluorescent microscopy [14]. Heyn et al labeled 231BR-EGFP cells with fluorescent micron-sized superparamagnetic iron oxide (MPIO) particles, and injected these cells into nude mice [15].

Single MPIO labeled breast cancer cells appeared initially after IC injection in the mouse as dark or hypointense voxels in the brain parenchyma on T2* weighted MRI that subsequently developed into metastases over 4 weeks [15]. Heyn et al reported that between 1-3% of the initially injected MPIO labeled cells remained in the brain to form the visible tumors on MRI [15]. Some of the labeled 231BR-EGFP cells were considered "dormant" based on the persistence of the hypointense signal in the same location of the brain over 28 days. Fluorescent microscopy demonstrated the presence of 231BR-EGFP cancer cells located within the cerebral vasculature in approximately the same location as the hypointense voxels on MRI. However, imaging studies with the brain seeking 231BR-EGFP breast cancer cell line have only been performed of the brain and therefore possible metastases in other tissues were not observed [15].

The goal of this study was to develop a rat model of brain metastases using the brain seeking breast cancer cell line 231BR that could be monitored with a clinical 3 tesla MRI. The 231BR cells were used because several reports have indicated that this breast cancer cell line was brain seeking and metastases were observed only in the brain by non-invasive imaging and histology [11-13,15]. The 231BR cells were stably transfected with firefly luciferase (231BRL) in order to determine the distribution of the breast cancer metastasis over time by bioluminescent imaging (BLI). The 231BRL cells were magnetically labeled with ferumoxides complexed with protamine sulfate [12-21] in order to monitor the early implantation of tumor cells in the brain and to determine the sensitivity of T2* weighted 3 tesla MRI to the labeled cells and the subsequent detection of multiple metastases. Most MR imaging studies of brain metastases have been performed in mice using high field strengths scanners (i.e., ≥ 7 tesla) because of the ability to obtain high spatial resolution and signal to noise as compared to images obtain using a clinical scanners [22,23]. Moreover, we employed multimodality imaging to direct the pathological examination from the unexpected areas of breast metastases that occurred following the IC infusion of the brain seeking 231BR breast cancer cell line [11].

## Methods

### Tumor cell line

The 231BR breast cancer cell line [11] was transfected with the mammalian expression vector pGL3-control (Promega Corporation, Madison, WI) using effectene reagent (Qiagen, Germantown, MD) according to the manufacturer's protocol to tag the cells with the firefly luciferase gene for bioluminescent imaging. The cells were co-transfected with pcDNA3.1 containing a neomycin resistance gene for the selection of clonal populations of luciferase expressing cells. Forty-eight hours after transfection cells were incubated in growth media containing 800 μg/ml G418 (Invitrogen, Carlsbad, CA) and single clones selected after 4 weeks in culture. Luciferase expression was confirmed using the Steady-Glo Luciferase Assay System according to the manufacturer's protocol (Promega Corporation, Madison, WI). The clone with the highest expression was used in the experiments described herein for the BLI tumor detection system. The cell line was cultured with DMEM growth media containing 10% fetal bovine serum and 1% penicillin streptomycin antibiotics (Invitrogen, Carlsbad, CA) at 37°C in room air with 5% CO_2_.

### Ferumoxides-Protamine Sulfate (FEPro) labeling of 231BRL cells

The 231BRL cells were labeled with the commercially available ferumoxides (Feridex IV^®^, 11.2 mg/ml, Bayer-Schering Pharmaceutical Inc, Wayne, NJ) contrast agent complexed to preservative free protamine sulfate (10 mg/ml, American Pharmaceuticals Partner, Schaumburg, IL) as previously described [13]. 231BRL cells were cultured until they reached 90% confluence. Ferumoxides (FE) and protamine sulfate (Pro) were mixed in fresh serum free RPMI 1640 medium (Biosource, Camarillo, Ca) at concentration ratio of FE:Pro of 100 μg/ml:6 μg/ml of media. The cells were incubated for two hours followed by overnight incubation with complete media. Labeled cells were washed 3 times with 10 unit/ml of heparinized PBS and trypsinized. Determination of average iron concentration per cell was done using a variable-field relaxometer (Southwest Research Institute, San Antonio, TX) as previously described [12,16,24].

### Cellular viability and proliferation

A trypan blue exclusion test was performed to determine the effect of FEPro labeling on the 231BRL cell viability. To determine the proliferation capacity of the FEPro labeled cells, MTS (3-[4,5-dimethylthiazol-2-yl]-5-[3-carboxymethoxyphenyl]-2-[4-sulfophenyl]-2H-tetrazolium, inner salt) cell proliferation assay (CellTiter 96^® ^AQ_ueous _One Solution, Promega, Madison, WI) was performed using the manufacturer's protocol.

All in vitro measurements were performed in triplicate.

### Animal model

This study was conducted under an approved Animal Care and Use Committee (ACUC) protocol at our institution. All procedures were performed using sterile technique. *In vivo *imaging studies including IC injection, BLI and MRI were performed with isoflurane gas anesthesia 2-3% mixed with 100% O_2 _by nosecone and body temperature was maintained 37°C. FEPro labeled or unlabeled 231BRL cells were suspended in 10 units/ml heparin in phosphate buffered saline (PBS). Tumor cells were introduced into the left ventricle under ultrasound guidance using a 14 MHz linear probe (Acuson Sequoia C256, Siemens Medical Solutions, Malvern, PA). Thirty-one female nude rats (NIH-*rnu *from Charles River, Wilmington, MA) at 6 to 8 weeks of age were divided into 3 groups for this study. Table 1 is a summary of the experimental design. Two cell doses were used in order to establish the minimal numbers of cells that would be required to establish this metastatic model in the nude rat. Group 1 consisted of 18 rats that received 3 × 10^6 ^FEPro labeled 231BRL cells by IC injection. Serial MRI and BLI studies were performed as part of a study and cohorts of animals were euthanized at specific time points (day 1,3 and weeks 1,2,3) post IC injection to determine the distribution of metastases in the brains at specific points in time. Group 2 rats (n = 8) were injected with 10^6 ^FEPro labeled 231BRL cells and were evaluated for the distribution of metastasis with confirmation on histological examination. Group 3 (n = 5) rats were injected 10^6 ^unlabeled 231BRL cells and served as controls for the imaging studies.

**Table 1:** Experimental Design

**Days**	**Group A rats (n = 18)**	**Group B rats (n = 8)**	**Group C rats (n = 5)**
Prior to infusion of cells	MRI baseline	MRI Baseline	MRI Baseline
Day 0 IC injection	3 × 10^6 ^FEPro labeled 231BRL	10^6 ^FEPro labeled 231BRL	10^6 ^unlabeled 231BRL
Day 1	MRI and BLI Euthanized 4 of 18 rats	MRI and BLI	MRI and BLI
Day 2	--	MRI and BLI	MRI and BLI
Day 3	MRI and BLI Euthanized 4 of 14 rats	MRI and BLI	MRI and BLI
Week 1	MRI and BLI Euthanized 5 of 10 rats	MRI and BLI	MRI and BLI
Week 2	MRI and BLI Euthanized 3 of 5 rats	MRI and BLI	MRI and BLI
Week 3	MRI and BLI Euthanized all rats	MRI and BLI	MRI and BLI
Week 4		- Euthanize 8 rats -	- Euthanize 5 rats -

### Imaging procedures

MRI scanning was performed on a clinical 3 tesla MRI unit (Intera, Philips Medical System, Netherlands, B.V.) with using a solenoid 4 cm radiofrequency receive only coil (Philips Research Laboratories, Germany) for rat brain. Physiological monitoring was performed with SAII MRI compatible unit (Small Animal Instruments Inc., Stony Brook, NY). The MR pulse sequences were as follows: T2-weighted (T2w) turbo spin echo (TSE) sequence, repetition time (TR)/echo time (TE) = 3200/60 ms, turbo spin echo factor 12, number of average (NAV) 8, field of view (FOV) 50 mm, slice thickness 0.5 mm, matrix 224 × 256, reconstructed resolution 100 × 100 μm, slice number 25; and a T2* multi echo gradient sequence (T2*w), TR/effective TE = 4560/28 ms, 15 echos, flip angle 30°, NAV 2, FOV 50 mm, slice thickness 0.5 mm, matrix 176 × 256, reconstructed resolution 200 × 200 μm. For contrast enhanced MRI studies gadopentetate dimeglumine (GdDTPA, 0.5 M, Magnevist, Bayer Schering Pharmaceuticals, NJ) at a dose of 0.3 ml/kg was injected through lateral tail-vein. Pre and post GdDTPA enhanced 3D T1-weighted (T1w) fast field echo (FFE) sequence were performed with TR/TE = 35/4 ms, flip angle 35°, NAV 8, FOV 50 mm slice thickness 0.5 mm × 25 sections and matrix 224 × 256 with a reconstructed in plane resolution 100 × 100 μm. The total MRI scanning time was less than 50 minutes per rat. For Group 2 and 3 rats, hyperintense masses on T2 weighted images on pre-euthanasia scans were manually counted and matched to regions of the brain in order to determine the distribution of metastases.

MRI studies were also performed in Group 2 and 3 rats to validate the presence of photon flux activity detected on BLI along the spinal cord of the animals. Sagittal MRI was performed of the spine as follows: T2w with TSE 3200/60 msec, field of view 50 mm, 0.5 mm slice thickness, matrix 224 × 172 reconstructed to 100 × 100 μm in plane resolution and T1w contrast enhanced FFE with 35.4/4.2 msec and flip angle of 35° with a field of view 50 mm and 0.5 mm slice thickness matrix size 224 × 172 reconstructed to 100 × 100 μm in plane resolution.

Bioluminescence imaging was performed using IVIS™ 100 system and analyzed with Living Image^® ^software (Xenogen, Alameda, CA). For *in vitro *studies, FEPro labeled and unlabeled 231BRL cells were prepared in a black 96 well plates (Corning Costar Company, Cambridge, MA) from 10^5 ^cells to 195 cells per well and D-luciferin Firefly (synthetic sodium salt monohydrate, Biosynth International, Inc, Naperville, IL) was added to each well at a concentration of 150 μg/ml. Total photon flux from each well was obtained over 1 minute and correlated to the numbers of cells per well. The *in vivo *BLI was performed following intraperitoneal injection of luciferin substrate at concentration of 150 mg/kg at 10-15 minutes following injection with a 3-minute acquisition time. The BLI photon flux in photons/sec/cm^2^/steradian was obtained and comparisons were made to the serially acquired images. The background photon flux was measured at the outside of the rat and was automatically subtracted.

### Histopathology

The rats were euthanized with an overdose of pentobartital Sodium (Nembutal, 50 mg/ml, Ovation Pharmaceuticals Inc., Deerfield, IL) and were perfused with heparinzed saline and 4% paraformaldehyde for histological examination. Brain, spinal cord, lung, liver, spleen, kidney, lymph node, bone marrow, and tumors in decalcified long bone and spine were harvested from animals. Six-micron thick sections were obtained and stained with hematoxylin and eosin of brain and other organs that had photon flux activity on pre-terminal BLI. Histological sections were of the brain were obtained in approximately the same plane as MRI to allow for imaging pathological comparisons. Consecutive sections were obtained for immunohistochemistry. IgG anti-human cytokeratin antibody (AE1/AE3, DacoCytomation, Denmark) and mouse IgG Vectastain^® ^Elite ABC Kit was used to detect the human breast cancer cells. Immunostaining kits were purchased from Vector laboratories (Vector Laboratories, Inc., Burlingame, CA). Sections were incubated with biotinylated secondary antibody for 30 min and enhanced with 3,3'-diaminobenzidine (DAB). Counterstaining was done with Vector^® ^Hematoxylin QS. Consecutive sections were stained with Perl's reagent (Prussian blue) for the presence of iron and counterstained with nuclear fast red as previously described [17,24,25]. Approximately 5-10 consecutive histological sections of the brain and selected tissues were analyzed and photographed under light microscopy (BX50F, Olympus Optical Co., LTD., Japan) for each rat. The images were processed using Adobe Photoshop 7.0 (San Jose, CA).

### Statistical analysis

In vitro study results were entered into standard spreadsheet software package and statistical significance was performed using two-tailed *t *test with P < 0.05. A regression analysis was performed to correlate cell number to the photon flux. All results are reported as the mean ± standard deviation.

## Results

### Cell labeling and in vitro analysis

Prussian blue (PB) staining proved homogenous high efficient labelling of 231BRL cells with FEPro (Additional file 1A). There were no significant differences between FEPro labeled and unlabeled cells in Trypan blue viability (i.e., 97.9% ± 1.96 versus 98.8% ± 1.98,) or for proliferation capacity as measured by MTS assay (i.e., Absorbance 1.244 ± 0.13 versus 1.158 ± 0.23). The average (n = 3 samples) intracellular iron content of was 10.7 ± 1.9 pg/cell for FEPro labeled cells and 0.3 ± 0.03 pg/cell for unlabeled 231BRL cells. There was a correlation between the cell number and photon flux intensity in vitro (Additional file 1B). Bioluminescence photon flux intensity of the 231BRL cells was not affected by FEPro labeling over the range of cell numbers evaluated with a minimum detection limit of 195 cells (Additional file 1C).

### In vivo imaging with histological correlation

**Figure 1:**
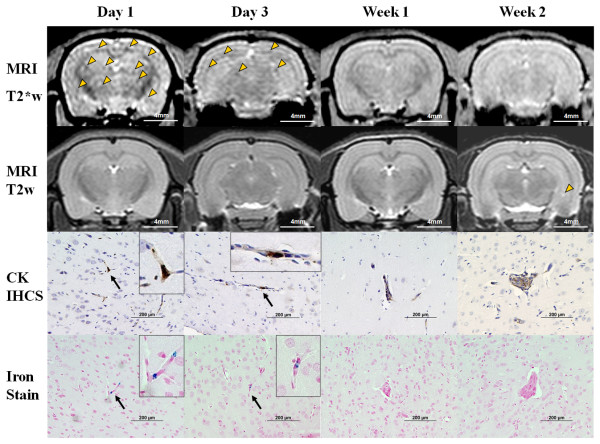
**In vivo cellular MRI with histological validation of brain metastases in group 1 rats**. Representative group 1 rats that received 3 × 10^6 ^FEPro labeled 231BRL cells with each column matched to the same animal. T2*-weighted images demonstrate diffuse brain metastasis of tumor cells as hypointense voxels on days 1 and 3 post intracardiac injection. Arrowheads mark some of the hypointense regions. Growing metastatic breast cancers were greater than 200-300 μm in size at week 2. T2-weighted image shows hyperintense tumor at left hippocampus at week 2 (arrowhead). Cytokeratin immunohistochemical staining (CK IHCS) of the brain showed tumor cells (i.e., brown) in the microvasculature of the brain at the early period (day 1-3, arrow) and growing mass at the later time points (week 1-2). Prussian blue iron staining were compatible findings to CK IHCS staining for tumor cells. Bar: MRI = 4 mm, histology = 200 μm.

MRI scans from group 1 rats that were euthanized at different time points is shown in Figure 1. T2*w images in the coronal plane demonstrate numerous hypointense (i.e., dark) voxels distributed throughout gray and white matter of 231BRL cells on days 1 and 3 post IC injection. The hypointense voxels were not observed on baseline scans in rats prior to receiving FEPro labeled cells (data not shown). Hypointense voxels were not detected on T2w images.

**Figure 2:**
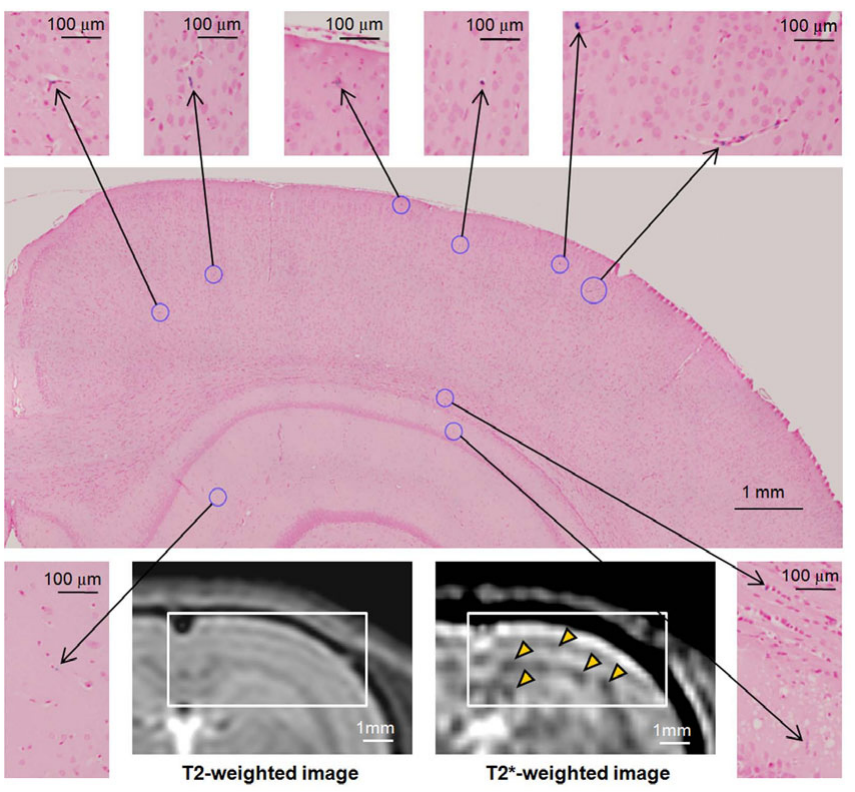
**MRI with histological correlation of group 1 rat**. Prussian blue staining of the coronal section of brain and MRI shows the distribution of ferumoxides labeled cells in the brain at day 1 post IC infusion of 3 × 10^6 ^cells. The T2*w image shows many hypointense spot due to metastatic tumor cells in the cerebral cortex and hippocampus. Inset in the MRI indicates the area photographed in Prussian blue staining (center). Although the differences in the slice thickness of the histological section (6 μm) and MRI (500 μm) precluded direct spatial co-localization of iron positive lesions, the distribution pattern of metastasis can be appreciated.

Pathological examination of rats euthanized on day 1-3 post cell infusion showed evidence of single or clusters of breast cancer cells in capillaries throughout the brain that were PB and cytokeratin positive (Figure 1). The location of PB positive cells in histology were in the approximate location as the hypointense voxels on T2*w MR imaging (Figure 2). One to two weeks post infusion of FEPro labeled cells brain sections were negative by Prussian blue stain in 50% of the Group 1 rats. By week 2 post-injection of labeled 231BRL cells, small (i.e., 200-300 μm) hyperintense (i.e., bright) regions newly observed in the brain on T2w images consistent with new tumor formation and not infarction since these lesions were not observed on previous T2 weighted images. Histological examinations of the brain sections from rats euthanized week 2 post IC injection revealed metastases ≥ 200 μm in size and no evidence of hemorrhage or infarction within the brain was observed. The differences in the slice thickness between the histological section (6 μm) and MRI (500 μm) precluded the direct spatial co-localization of FEPro containing cells to the hypointense voxels on the T2*w image, the distribution of breast cancer cells appeared in similar areas of gray and white matter based on anatomical landmarks from MRI and histological sections. In order to reduce the Gibbs (ringing) image reconstruction truncation artefact [26] observed on the coronal plane T2w and T2* w images in the group 1 rats, MRI studies performed in the Group 2 and 3 rats were acquired in the oblique axial plane.

**Figure 3:**
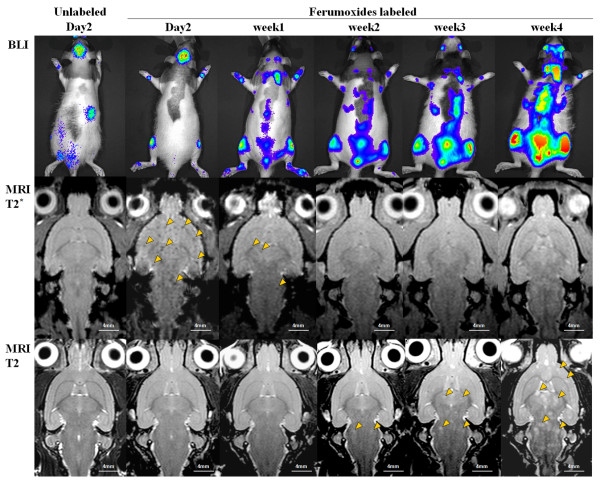
**Bioluminescence imaging and MRI obtained from representative a rat from Group 2 and Group 3**. First column contains representative images from Group 3 rats that received unlabeled breast cancer cells. Columns 2-6 (left to right) contain serial scans from Group 2 rat that received 10^6 ^FEPro labeled 231BRL cells. At day 2, BLI demonstrate intense photon flux from the brain from both groups of animals. T2*-weighted images demonstrate the presence of numerous hypointense voxels containing labeled cells (middle row, arrowhead). Arrows on T2-weighted images indicates growing metastatic breast cancer that can be seen as early as 2 weeks after infusion of cells.

Figure 3 contains examples of serial BLI and oblique axial MRI scans from one of the group 2 and group 3 rats. Serial BLI revealed intense photon flux activity originating from the brain within the first 2 days after injection of 231BRL cancer cells in group 2 and 3 rats (Figure 3). T2*w images performed on day 2 post FEPro labeled 231BRL cell injection shows numerous hypointense voxels distributed in the cerebrum, brain stem and upper cervical regions in Group 2 rats. Hypointense voxels were not detected on T2*w images from Group 3 at any point following infusion of cells. Region of interest from the head of group 2 and 3 rats revealed an increase in photon flux activity on Day 2 that rapidly decreases by Day 3 through week 1 post IC injection of breast cancer cells. This rapid decrease in photon flux between day 3 through week 1 to near background is consistent with the decrease in number of hypointense voxels on T2*w images and clearing of FEPro labeled 231BRL cells from brain. This change in signal intensity on MRI occurring days after infusion of FEPro labeled cells indicates that either cell metabolized or diluted ferumoxides through multiple cell divisions or only minority of the breast cancer cells remained in the brain, marginated into the parenchyma, proliferated and formed metastases. The disappearance of hypointense voxels during this period of time occurred in 100% of Group 2 rats. Between weeks 1 and 3 post infusion of FEPro labeled cells, there was limited evidence of tumor cell proliferation detected by BLI (Figure 3). Photon flux decreased from 5.91 × 10^6 ^photon/sec to 1.03 × 10^5 ^photon/sec levels between day 2 and week 1 post infusion of cells with a rapid rise in bioluminescent activity at 3 weeks to above day 1 levels (> 10^7 ^photons/sec) and was consistent with tumors detected by MRI and on histological examination (Additional file 2).

T2w images between 2-4 weeks post IC injection of 231BRL revealed multiple metastases in the brain. The distribution of brain metastases in groups 2 and 3 rats by week 3-4 was similar to that observed in the group 1 animals on the imaging studies. The majority of hyperintense brain metastases on T2w were located in the cerebral cortex (100%), thalamus and hypothalamic regions (92%), hippocampus and pons/medulla (85%) and tumors were less commonly found (> 55%) in the olfactory bulb, cerebellum and midbrain regions.

### Histological findings in metastases in the body

**Figure 4:**
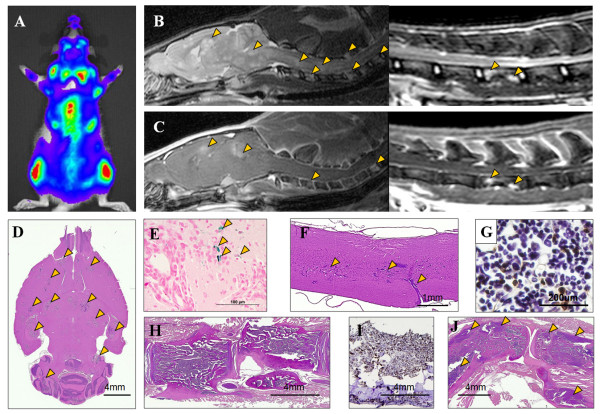
**Central Nervous System and skeletal involvement by breast cancer**. A) Bioluminescence image of rat at week 3 shows high photon flux activity from the brain, spine, and joints. (B) Sagittal T2w MRI and (C) contrast enhanced T1w MRI show hyperintense lesions on the brain, spinal cord, and vertebral bodies (arrowheads). Histological section of brain (D) and spinal cord (F) with hematoxylin and eosin (HE) staining from group 2 rat euthanized at week 4 reveals numerous metastases (arrowheads). E) Prussian blue staining of the consecutive brain section from D shows few isolated iron positive cells near the tumor (arrows). H) Thoracic spine with tumor infiltration on HE stain. Cytokeratin immuno-histochemical staining of the bone marrow aspirates (G) and Spine (I) is positive for tumor. J) Knee joint metastasis with extraskeletal involvement is seen (arrowheads) on HE stain.

The distribution of breast cancer metastases determined from histology and imaging for the three cohorts of animals was summarized on Table 2. Labeling the 231BRL cells with FEPro did not alter the breast cancer cells ability to produce metastases in the brain. The development of metastatic breast cancer was organ dependant. Figure 4 contains representative BLI and MRI of the distribution of metastases in the spinal cord and bones. Prussian blue staining was usually negative for iron one week after infusion of FEPro labeled cells, however isolated PB positive breast cancer cells could be detected in the brain parenchyma at euthanasia in 5 of 8 of the group 2 rats (Figure 4E). All rats presented metastatic breast cancer cells in the bone marrow and multiple lytic bone lesions in axial and appendicular skeleton as early as week 2. Both spinal cord and vertebral body metastases were observed in this model along with tumor cell infiltrations in and around joint spaces (Figure 4J).

**Table 2:** Distribution of metastatic tumor determined on histology and imaging

	**Group 1**					**Group 2**	**Group 3**
			
**%**	**Day 1 (n = 4)**	**Day 3 (n = 4)**	**Week 1 (n = 5)**	**Week 2 (n = 3)**	**Week 3 (n = 1)**	**(n = 8)**	**(n = 5)**
Brain^a^	0	0	80	100	100	87.5	80
Spinal Cord^a^	0	0	80	100	100	87.5	80
Lung^a^	0	0	0	20	0	50	60
Liver^a^	0	0	20	33.3	100	0	0
Kidney^a^	0	0	60	100	100	87.5	60
Lymph Node^a^	0	0	100	100	100	100	100
Heart	0	0	0	0	0	0	0
Bone^b^	100	100	100	100	100	100	80

Note. a. Tumor cells within the vasculature or tissue on day 1 and 3 were not counted.

b. Positive tumor cells detected by immunohistochemical staining of bone marrow sample were considered as positive lesion on group 1 rats. Group 2 rats underwent histological examination of long bone and spine for bone tumor determination. In group 3 rats, bone tumor lesions were determined on MRI and BLI.

All rats had evidence of tumor cell infiltration in the lymph nodes with distortion of nodal architecture. Lung, liver and renal metastases were found in animals euthanized at later time points however mass lesions (> 200 μm) were rarely seen (Additional file 3). In the spleen, breast cancer cells were diffusely disseminated throughout red and white pulp but no discrete mass lesions were found.

## Discussion

The development of a relevant large rodent model of brain metastases that can be monitor using relevant non-invasive techniques is needed to investigate the early distribution pattern of tumors and translate the imaging approaches to the clinic [8]. The major finding of this study was the documentation using multimodality imaging approach of the development of tumor metastases model in the nude rat using the brain seeking 231BRL cell line. Bioluminescent imaging and MRI demonstrated primarily brain and bone metastases from this brain seeking breast cancer cell line. The 231BR breast cancer cell line was chosen for this study since it reported only produced brain metastases [11-13,15] and we wanted to determine if we could develop model in large rodent and possibly go on to use bioluminescent activity in the brain as an outcome measure for future treatment. The surprising finding was that all of the breast cancer 231BR cell lines resulted in bone metastasis that was not previously described and missed on pathological examination. The results of this study strongly indicate the need of noninvasive whole body imaging when developing a new animal model. Jenkins et al [27] reported that following the transfection of MDA-MB-231 cells with firefly luciferase, the pattern of metastases was altered compared to the parent cell line and resulted in tumors in bone, brain, liver, lungs, lymph nodes, and kidneys. In this study, the fluc gene transfected 231BR cells also caused bone metastases in a similar distribution as previously reported in mice [27]. In addition, the distribution of brain metastases in the rat model was similar to previous report in mice injected with green fluorescent protein transfected 231BR cell line [12]. Of note, we have IC infused 231BR or 231BRL cells into nude mice and have observed development of bone metastases on BLI and micro CT scan (Additional file 4). These results indicate that the introduction of fluc into the cell genome appears to have altered the propensity of selective metastases of the 231BR cells to just the brain. Differences in genotype expression of the 231BRL cells at the various metastatic sites would be impressive but highly unlikely or significant and would require gene chip analysis that is beyond scope of this work. The findings in this study support the importance of multimodal imaging in the development and evaluation of cell lines intended to model clinical disease or home to a specific target tissue.

In the current study, photon flux activity was not visible originating from the brain in 75% of the Group 1 rats between weeks 1 to 2 post IC injection of cells, even though metastases were detected over 80% of rats on histology and MRI. The region of interest analysis of the photon flux from the heads of the group 2 rats between day 3 and week 1 shows an abrupt decrease toward background level (Additional file 2). The decrease in photon flux intensity in the rat brain between day 3 and week 1 post IC injection may be attributed to a change in pigmentation of the hair and skin of the animals [28], tumor cells being cleared from brain vasculature, areas of tumor hypoxia or poor delivery of luciferin to the tumors. Although the measured photon flux intensity from the brain region at week 3 is elevated compared to day 1 post infusion of 231BRL cells (Additional file 2), brain metastases detected on T2w images were not always present on BLI (Figure 3). Extrapolation from the in vitro photon flux activity versus numbers of the 231BRL cells injected (Additional file 1) would estimate that approximately 8.5% of the initially IC injected 10^6 ^tumor cells were located in the head on day 2. Between day 3 and week 1, the photon flux decreased and was estimated to be approximately 0.6% of the cells that remained or survived in the rat brain ultimately going on to form metastases. Heyn et al [15], reported that between 1-3% of the initially injected MPIO labeled cells remained in the mouse brain and went on to form the metastases at 4 weeks post infusion of cells. The difference between these two studies probably can be contributed to the size of the animals, the fluorescent versus luminescent labels in breast cancer cells and the in vivo optical imaging devices used and that MPIO are not metabolized in cells as compared to ferumoxides that dissolves in endosomes [29,30]

The disappearance of hypointense voxels (i.e., voxels becoming isointense to surrounding brain) on the T2*w images by week 1 post IC injection of cells can be due to multiple factors including dilution of the FEPro label in rapidly proliferating cells [17], iron metabolism [29,31] and/or the cells became apoptotic or died and were cleared from the vasculature before marginating into the parenchyma. Prussian blue stain of the brain revealed rare cells with intracellular iron in the cortex of 50% and 62.5% of group 1 and 2 rats euthanized at 2-4 weeks, respectively. The small numbers of PB positive cells were not detected using T2* weighted images probably because of the spatial resolution and image contrast (Figure 4E). The finding of isolated PB positive cells on histology would suggest the need for quantitative imaging approaches such as T2* maps that can be possibly correlated to amount of iron present in the brain [32,33]. Heyn et al [34] reported the detection of a single MPIO labeled breast cancer cells in the brains of mice with a modified clinical 1.5T MR scanner using acquired with a voxel size of 39 × 39 × 100 μm at 4 weeks post infusion of cells. The apparent difference in being able to detect single dormant breast cancer cells at > 1 week post infusion between the latter study and current study (voxel size 100 × 100 × 500 μm) maybe due to the increased susceptibility effect generated by the MPIO versus the SPIO nanoparticles, smaller voxel size produced by MRI hardware modification and the MPIOs are not metabolized by the cell. The combination of quantitative T2* MR imaging performed prior to and post infusion of magnetically labeled cell should provide investigators with the ability to detect subtle changes in T2* maps that would indicate the persistence of SPIO in cells in the brain [35] that may be used in cell therapy trials.

MRI and BLI provided serial non-invasive assessment of the formation of metastases over time in the same cohort of animals. In the current model, 84.6% of all the animals developed brain metastases in 4 weeks when 10^6 ^cells were administered in 6 week-old nude rats. The distribution of 231BRL metastases in the rat brain on MRI is similar that has recently been observed on histological examination of the mouse brain following IC injection of the 231BR-EGFP by Fitzgerald et al [12]. In the current study, metastases in the olfactory bulb and cerebellum were not as common as observed in the mouse brain [12]. This difference may be contributed to MRI inability to clearly delineate micro-metastases because of image contrast on T2w images. The intent of this study was to develop a model of breast cancer metastases in the rat brain using a cell line that had been shown to produce brain metastases that could be detected using a clinical 3 tesla MRI unit. BLI demonstrated that IC injection of this brain seeking fluc transfected 231BR breast cancer cell line resulted in the development of metastases in the skeleton. Follow-up high-resolution MRI studies of these areas confirmed the presence of metastatic breast cancer. By using this multimodality approach, spinal cord metastases were also detected by MRI (Figure 4B-C) that had previously not been identified in mouse model studies using the 231BR cell line [11,14,15]. Of note, spinal cord metastasis represents about 8.5% of CNS metastasis and affects 0.1 to 0.4% of cancer patients [36]. The detection of spinal cord metastases by MRI underscores the importance on using multimodality imaging techniques to evaluate experimental models of metastatic disease.

## Conclusion

In this study, serial BLI and MRI were used to track the temporal and spatial distribution of breast cancer from the 231BRL cell line and the formation of metastases in the rat brain. MRI detected brain metastases in the rat brain 2-4 weeks following the IC injection of 10^6 ^231BRL cells in this highly reproducible model and findings confirmed on histological examination. Multimodality imaging detected the presence of metastases in the brain and spinal cord, bone and other internal organs during the disease course demonstrating the important role of in vivo imaging in the development of an experimental model. This rat model and non-invasive clinically relevant MRI techniques and BLI should be useful for the development of novel targeted drug, cellular and molecular therapies for the treatment of metastatic breast cancer.

## Competing interests

The authors declare that they have no competing interests.

## Authors' contributions

HTS conceptualized, designed, and supervised the overall study; performed MRI and bioluminescence imaging experiments, performed animal modeling and histology, analyzed the data, interpreted the overall study results and prepared the manuscript. EKJ assisted the entire animal imaging experiments and performed immunohistochemistry, prepared the manuscript, and contributed to the design of the study. BKL assisted in vivo MRI experiments and processed the MRI data. WL assisted the interpretation of MRI data. JG assisted animal experiments and performed histology. BK assisted intracardiac injection of tumor cells to the nude rat. DD performed real time ultrasonography guiding intracardiac injection in animal modeling. DP performed luciferase gene trasnfection to MDA-MB-231BR cell line. JAF equally contributed to conceptualize, design and supervise the overall study and prepared manuscript.

## Supplementary Material

Additional file 1 **Validation of FEPro labeling and bioluminescence photon flux intensity**. A) Prussian blue staining (blue color) of the FEPro labeled human breast cancer cells proved homogenous intracellular labeling of the cells. Inset shows unlabeled control cells. B) The number of MDA-MB-231BRL breast cancer cell and bioluminescent signal intensity was linearly correlated (R^2 ^= 0.9997). Bioluminescence activity was measured as total photon flux for each well. C) Well plate measurement of bioluminescent intensity of FEPro labeled and unlabeled 231BRL cells show no difference. D) Table shows actual photon count from well experiment of (C). E) Average photon count of 4 session of triplicate experiment. No statistical significance of difference of photon count before and after FEPro labeling was proved. Click here for file

Additional file 2 **Region of interest photon flux analysis from the brain and whole body in group 2 rats**. BLI on Day 2 shows a peak in the photon flux activity originating from the brain whereas activity from the body was at its minimum from days 2-3 post infusion of the 231BRL cells. Photon flux from the body increases rapidly in the body from weeks 1-3 and has greater number of counts as compared to the brain. Whole body does not include photon flux from brain. Click here for file

Additional file 3 **Organ involvement of the metastatic tumor**. Hematoxylin and Eosin (HE) and cytokeratin (CK) staining of the major internal organs are shown. Diffuse breast cancer cell infiltrations were present in the lymphoid tissue of the lung, lymph nodes and spleen. Hepatic periportal tumor cell infiltration in the liver (double arrow in CK in Liver) was frequently observed in the rats. Hepatic sub-capsular metastatic lesions were rarely found (arrow). Tumor cell infiltrations in renal glomeruli were also frequently observed (arrows). Click here for file

Additional file 4 **Bone metastases produced by brain seeking breast cancer cell in the nude mouse**. A) An example of one of the nude mice (n = 6) that received 1×10^5 ^brain seeking luciferase transfected MDA-MB-231BR cells reportedly the brain seeking breast cancer cell line by intracardiac injection. Three weeks post injection of cells bioluminescence images show photon flux activity over the spine (arrows), head, scapular, lung and kidney (arrowheads). B) Three dimensional volume rendered image by using MicroCAT II micro CT scanning system (Siemens Preclinical Solutions, Knoxville, TN) of nude mouse at 5 week from intracardiac injection of 1×10^5 ^MDA-MB-231BR cells. Multiple osteolytic lesions on the proximal shoulder, scapular, knee, and spine are seen (arrowheads). Click here for file
