# Supplementary figures and images for: Rat model of metastatic breast cancer monitored by MRI at 3 tesla and bioluminescence imaging with histological correlation
Source: J Transl Med. 2009 Oct 20;7:88. doi: 10.1186/1479-5876-7-88 (PMC2774309; doi:10.1186/1479-5876-7-88)

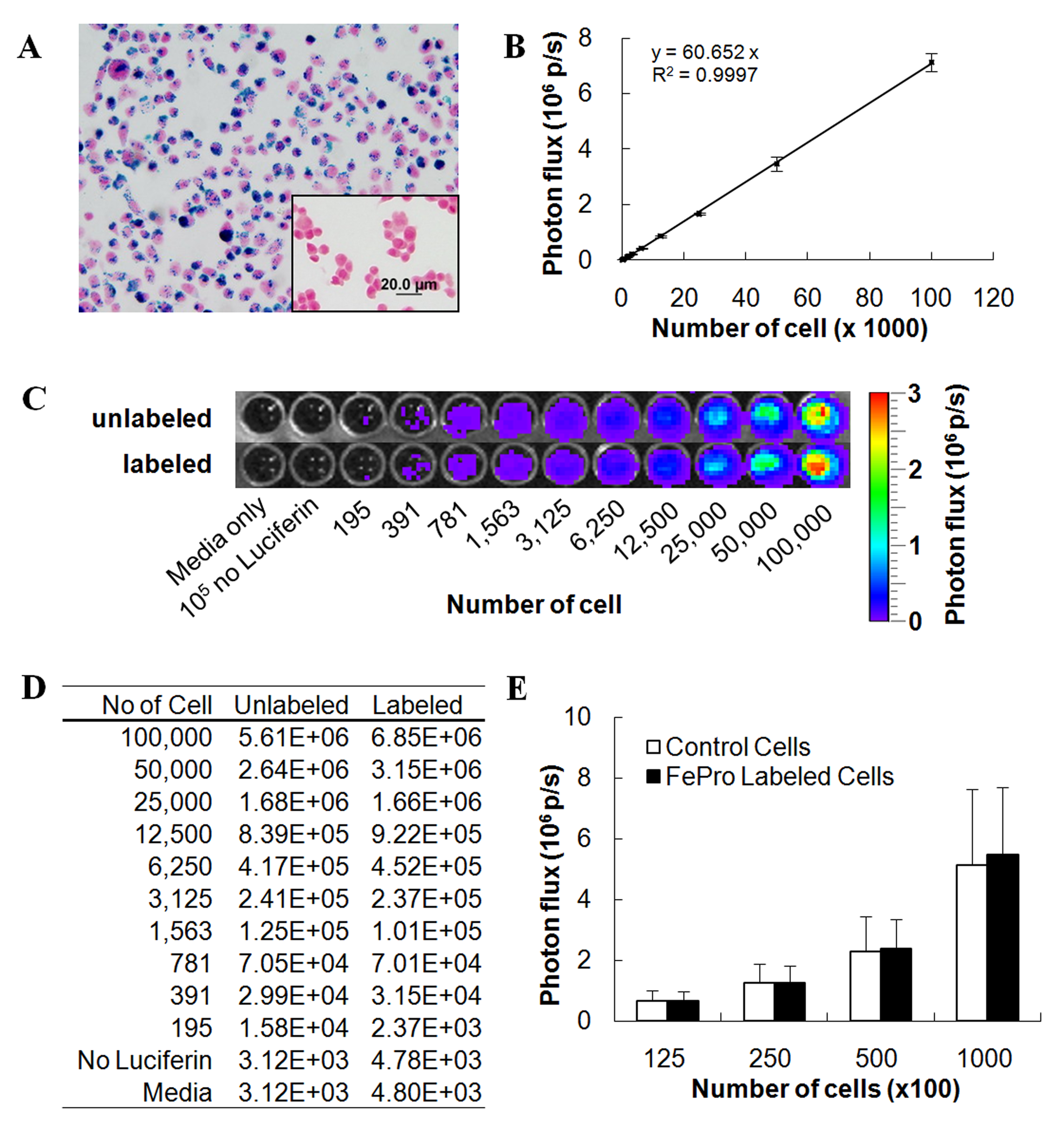

Supplement: Additional file 1 — Validation of FEPro labeling and bioluminescence photon flux intensity. A) Prussian blue staining (blue color) of the FEPro labeled human breast cancer cells proved homogenous intracellular labeling of the cells. Inset shows unlabeled control cells. B) The number of MDA-MB-231BRL breast cancer cell and bioluminescent signal intensity was linearly correlated (R2 = 0.9997). Bioluminescence activity was measured as total photon flux for each well. C) Well plate measurement of bioluminescent intensity of FEPro labeled and unlabeled 231BRL cells show no difference. D) Table shows actual photon count from well experiment of (C). E) Average photon count of 4 session of triplicate experiment. No statistical significance of difference of photon count before and after FEPro labeling was proved. [file 1479-5876-7-88-S1.TIFF]

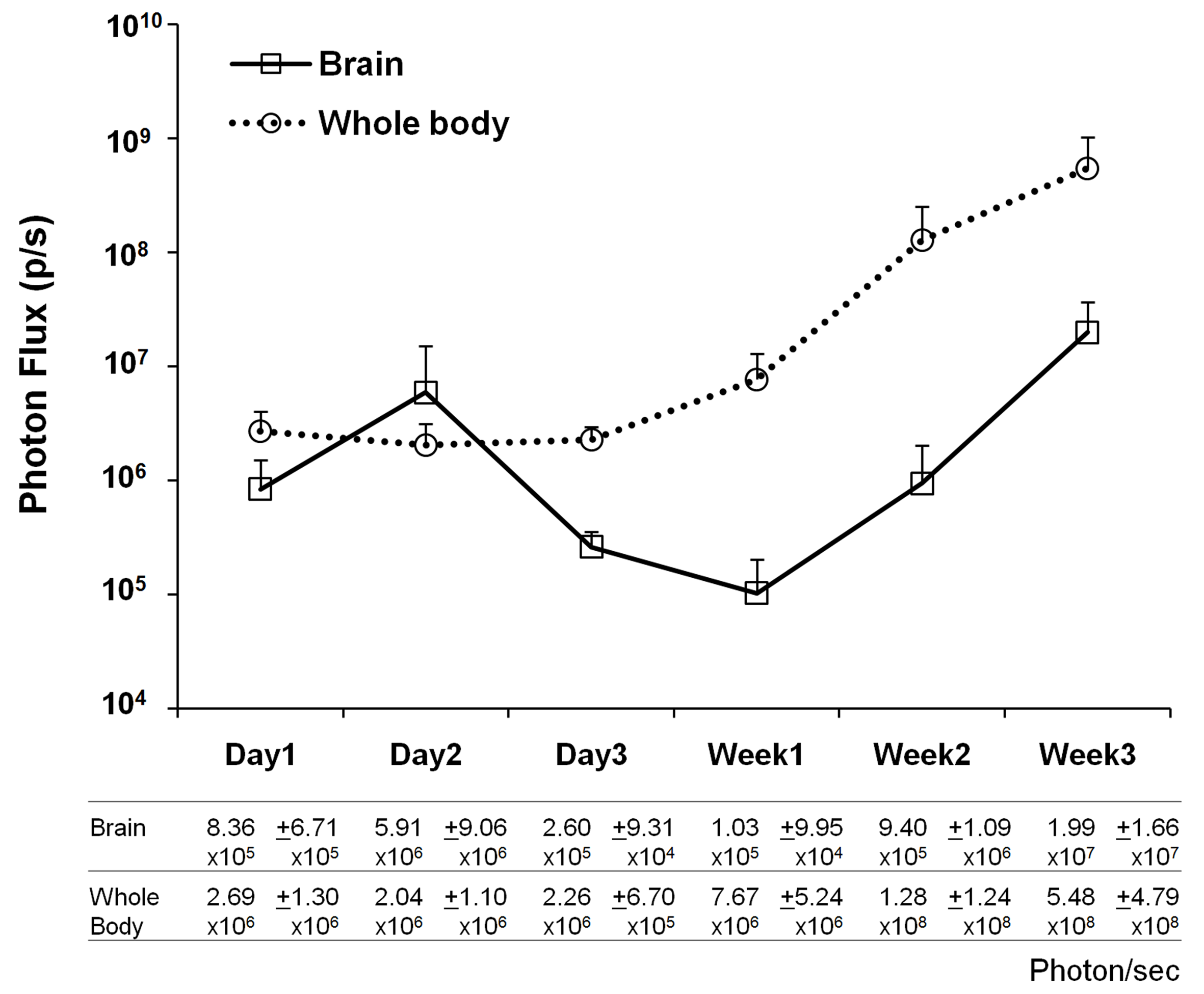

Supplement: Additional file 2 — Region of interest photon flux analysis from the brain and whole body in group 2 rats. BLI on Day 2 shows a peak in the photon flux activity originating from the brain whereas activity from the body was at its minimum from days 2-3 post infusion of the 231BRL cells. Photon flux from the body increases rapidly in the body from weeks 1-3 and has greater number of counts as compared to the brain. Whole body does not include photon flux from brain. [file 1479-5876-7-88-S2.TIFF]

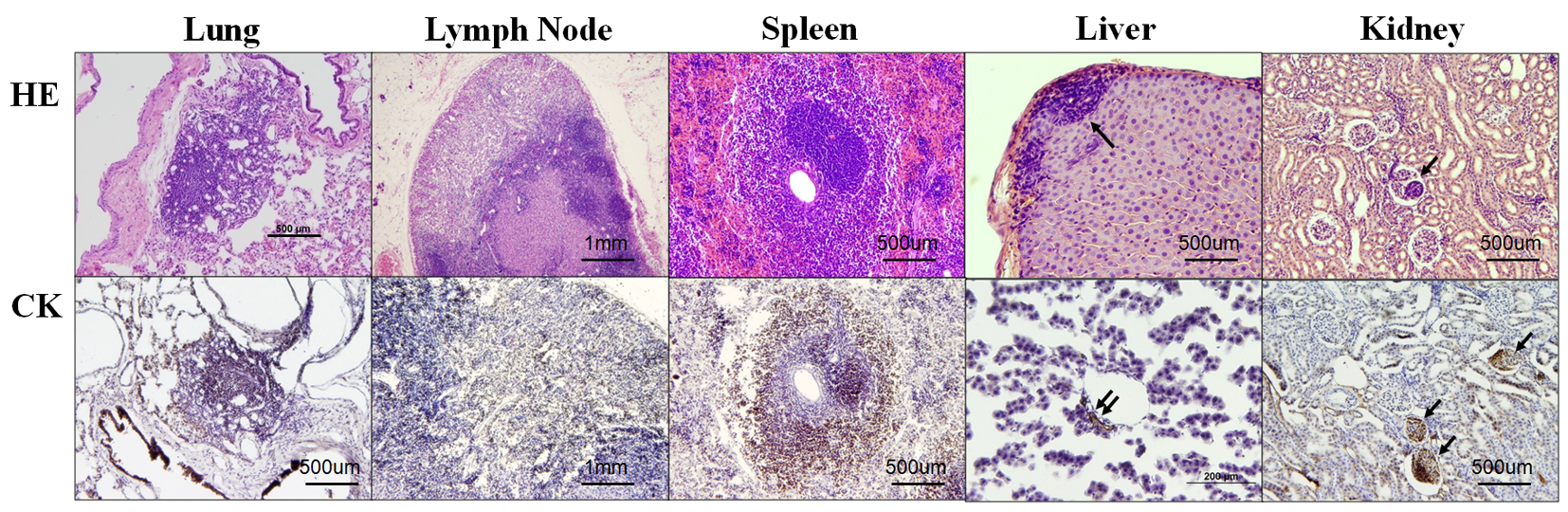

Supplement: Additional file 3 — Organ involvement of the metastatic tumor. Hematoxylin and Eosin (HE) and cytokeratin (CK) staining of the major internal organs are shown. Diffuse breast cancer cell infiltrations were present in the lymphoid tissue of the lung, lymph nodes and spleen. Hepatic periportal tumor cell infiltration in the liver (double arrow in CK in Liver) was frequently observed in the rats. Hepatic sub-capsular metastatic lesions were rarely found (arrow). Tumor cell infiltrations in renal glomeruli were also frequently observed (arrows). [file 1479-5876-7-88-S3.TIFF]

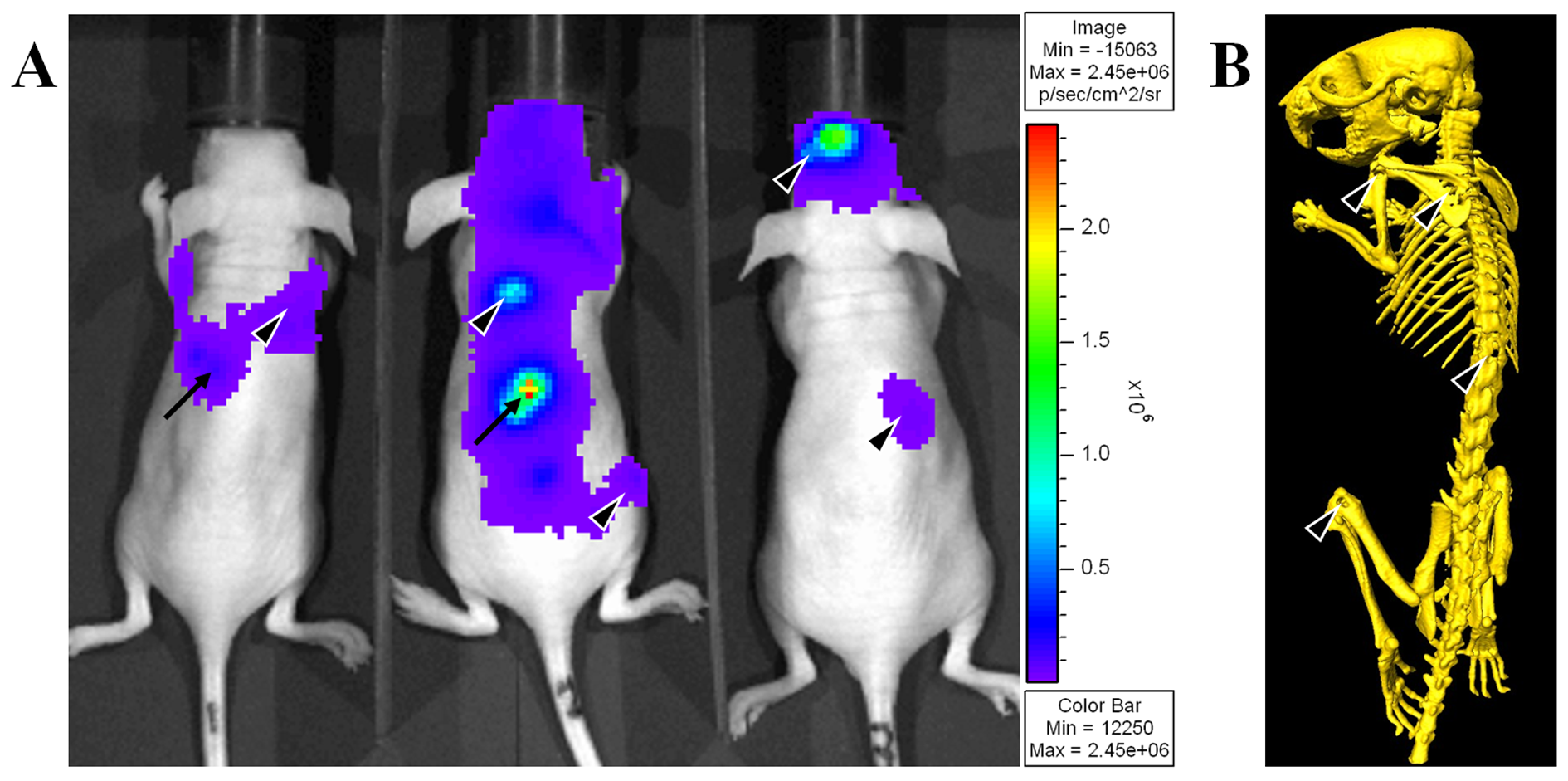

Supplement: Additional file 4 — Bone metastases produced by brain seeking breast cancer cell in the nude mouse. A) An example of one of the nude mice (n = 6) that received 1×10^5^ brain seeking luciferase transfected MDA-MB-231BR cells reportedly the brain seeking breast cancer cell line by intracardiac injection. Three weeks post injection of cells bioluminescence images show photon flux activity over the spine (arrows), head, scapular, lung and kidney (arrowheads). B) Three dimensional volume rendered image by using MicroCAT II micro CT scanning system (Siemens Preclinical Solutions, Knoxville, TN) of nude mouse at 5 week from intracardiac injection of 1×10^5^ MDA-MB-231BR cells. Multiple osteolytic lesions on the proximal shoulder, scapular, knee, and spine are seen (arrowheads). [file 1479-5876-7-88-S4.TIFF]
